# Decimal Arrangement

**Published:** 1881-04

**Authors:** 


The Decimal Arrangement.—Which we proposed in our February issue for the consideration of our brethren is far more simple than the French metric, and is equally convenient, and intended to harmonize with our computation of money and its values, viz: To apportion the doses of medicines to mills, cents, dimes, dollars and eagles. Those names, or their symbols, might be retained or others substituted of equal value if thought advisable. It would be very easy to adopt the money symbols, and use as e. g.: M. from i to ix for mills; C. from i to ix for cents; D. from i to ix for dimes; $. from i to ix for dollars, and E. from i to ix for eagles. The sign for half dimes, half dollars, etc., might be the same, ss., as at present. Let the mill represent half a grain, and thus a secure basis would be had to begin with. e. g.: Calomel, might be written for thus, calomel, C.ij.; for grs., x.; Quinia, C.j.; for grains, v.; Opium, M.ij.; for gr., j., and so on indefinitely. These examples will suffice to show the great simplicity of *our* proposed *decimal* system. In addition to this every one handling, reading or preparing such a prescription, or receipe, would be perfectly familiar with the signs, and their values throughout our entire country. No such familiarity with the Metric system could be expected from every one interested in writing or preparing a prescription for some time to come; as it would require a generation, at least, for such acquaintance with that system —*decimal*, though *it* might be regarded, as to secure accuracy in placing and reading ciphers and decimal points in such a manner as to *prevent* misconceptions or making of mistakes by the apothecary! The loss of a single life from the want of the necessary acquaintance with those two points so essential to a perfect understanding of the proportions of ingredients, should alone be sufficient to prevent its adoption; and where such knowledge of them is wanting to insure accuracy, it is obvious that not one only, but many lives would be certainly sacrificed, ere such intimate acquaintance and familiarity with them could be generally obtained, as to insure certainty in these respects, and thereby the avoidance of fatal mistakes. But sufficient for the present.

